# Molecular Classifications and Candidate Biomarkers for Personalizing BCG Therapy in Non-Muscle-Invasive Bladder Cancer

**DOI:** 10.3390/biomedicines14092098

**Published:** 2026-09-17

**Authors:** Airat Bilyalov, Andrey Kotov, Natalia Bodunova, Maxim Zingerenko, Igor Khatkov, Ludmila Zhukova

**Affiliations:** 1 Loginov Moscow Clinical Scientific Center, 111123 Moscow, Russia; 2 Life Improvement by Future Technologies (LIFT) Center, 121205 Moscow, Russia

**Keywords:** non-muscle-invasive bladder cancer, Bacillus Calmette–Guérin, BCG-unresponsive disease, molecular classification, predictive biomarkers, tumor microenvironment, immune checkpoint inhibitors, bladder-preserving therapy

## Abstract

Intravesical Bacillus Calmette–Guérin (BCG) remains the standard adjuvant treatment for intermediate- and high-risk non-muscle-invasive bladder cancer. However, 30–50% of patients fail to respond or experience recurrence within two years, while clinical and pathological risk models have limited accuracy in predicting individual response. This narrative review summarizes molecular classifications and candidate biomarkers associated with BCG response and assesses their potential clinical utility, based on a focused search of PubMed, Scopus, and Web of Science through July 2026, supplemented by ClinicalTrials.gov, regulatory documents, and current guidelines. Transcriptomic classifications, including UROMOL2021, BCG Response Subtypes (BRS), MSP888, and subtypes defined in stage T1 disease, identify tumor phenotypes associated with differences in recurrence, progression, and outcomes after BCG. *BRS3* is characterized by basal-squamous and immunosuppressive features and is associated with shorter recurrence-free and progression-free survival. Genomic alterations, including *FGFR3* and *TERT* promoter mutations and *APOBEC* signatures; DNA methylation markers such as *GATA2*, *TBX3*, and *GPR158*; and tumor microenvironment features, including regulatory T cells, exhausted CD8+ T cells, and PD-L1 expression, have been associated with outcomes in individual cohorts but remain unvalidated as treatment-specific predictors. Bladder-preserving options for BCG-unresponsive disease include pembrolizumab, nadofaragene firadenovec, nogapendekin alfa inbakicept-pmln with BCG, and the gemcitabine intravesical system. Randomized trials adding checkpoint inhibition to BCG in BCG-naive disease have reported inconsistent results. No molecular biomarker is currently validated for treatment selection. Molecular profiling remains investigational and may complement, but not replace, established clinical risk assessment. Clinical implementation requires prospective biomarker-stratified trials with standardized definitions, assays, and endpoints.

## 1. Introduction

Bladder cancer ranks 9th in global cancer incidence and 13th in mortality, according to GLOBOCAN 2022 [1]. Approximately 75% of newly diagnosed cases are non-muscle-invasive, making it the primary patient population for adjuvant intravesical therapy. In terms of somatic mutation frequency, urothelial carcinoma is among the malignancies with the highest somatic mutation burden, alongside melanoma and non-small-cell lung cancer. According to The Cancer Genome Atlas (TCGA), a substantial proportion of somatic mutagenesis is attributable to the activity of the APOBEC family of cytidine deaminases, which account for approximately 65% of all somatic substitutions. An additional feature is the high frequency of alterations in chromatin-modifying genes, in particular *KDM6A*, *ARID1A*, *KMT2D* and *CREBBP* [2].

Intravesical immunotherapy with the live-attenuated Bacillus Calmette–Guérin (BCG) vaccine, first used for superficial bladder tumors in 1976, has become the standard of adjuvant treatment for intermediate- and high-risk non-muscle-invasive bladder cancer after nearly 50 years of clinical use [3,4]. Despite its long clinical history, its efficacy is limited. Estimates indicate that 30% to 50% of patients do not initially respond to therapy or experience disease recurrence within two years of completing the induction course. A clinically relevant proportion of patients with stage T1 disease subsequently progress to muscle-invasive bladder cancer [5]. Existing prognostic models developed by the European Organization for Research and Treatment of Cancer (EORTC) and the Spanish Urological Oncology Club (CUETO), as well as the European Association of Urology (EAU) stratification system updated in 2021, are based on clinical and morphological features such as stage, grade, tumor size and number, the presence of concomitant carcinoma in situ, and patient age. However, these models have limited predictive accuracy for the effectiveness of BCG therapy specifically, as patients within the same risk group often exhibit diametrically opposed clinical outcomes. This heterogeneity reflects the biological heterogeneity of the disease, which cannot be fully characterized by standard histopathological parameters.

The advent of next-generation sequencing, whole-transcriptome and multi-omics analyses, and single-cell and spatial transcriptomics have significantly expanded our understanding of urothelial cancer biology. Identification of molecular subtypes, characterization of the mutational landscape, and analysis of the tumor microenvironment have enabled more detailed evaluation of treatment-relevant tumor features.

This review summarizes molecular classifications and candidate biomarkers associated with response to BCG and examines their potential clinical application. The review first outlines the biological and clinical framework of BCG response, then examines molecular classification systems and candidate predictors, and finally discusses current and investigational treatment strategies and the prospective integration of molecular data into clinical decision-making.

## 2. Materials and Methods

A focused literature search was conducted in PubMed, Scopus, and Web of Science for publications available up to July 2026. ClinicalTrials.gov, the official websites of the US Food and Drug Administration, and the European Association of Urology guidelines were additionally consulted to identify ongoing clinical trials, regulatory decisions, and current recommendations. Search terms included combinations of “non-muscle-invasive bladder cancer”, “BCG therapy”, “BCG response”, “BCG failure”, “BCG-unresponsive”, “molecular classification”, “transcriptomic subtype”, “genomic biomarker”, “DNA methylation”, “tumor microenvironment”, “immune checkpoint inhibitor” and “intravesical therapy”. Original studies, clinical trials, consensus classifications, guidelines, and regulatory documents relevant to molecular predictors of BCG response and treatment strategies were prioritized. Reference lists of selected publications were also reviewed to identify additional relevant studies. Because this work was designed as a narrative review, no formal systematic-review protocol, quantitative meta-analysis, or standardized risk-of-bias assessment was applied.

## 3. Biological and Clinical Framework of BCG Therapy

### 3.1. Biological Basis of Intravesical BCG Therapy

The search for molecular determinants of BCG response requires consideration of its antitumor mechanisms. Following instillation, BCG attaches to the urothelium, partly through interactions with fibronectin, and can be internalized by urothelial cells, tumor cells, and resident immune cells. These events initiate local innate and adaptive immune responses and may also contribute to direct tumor-cell death [6,7].

The antitumor response to BCG is predominantly Th1-oriented and involves the secretion of IL-2, IL-12, IFN-γ, TNF, and TNF-β. Activation of dendritic cells is accompanied by antigen presentation to CD4-positive T lymphocytes, which in turn ensure the maturation of cytotoxic CD8-positive T cells and the activation of macrophages and natural killer cells. Activation of this immune cascade leads to the destruction of tumor cells both through a direct cytotoxic mechanism and through the formation of specific antitumor immune memory [7].

Intravesical BCG can induce trained immunity, characterized by functional and epigenetic reprogramming of circulating monocytes and enhanced responses to heterologous stimulation [8]. This mechanism may contribute to the effects of repeated BCG exposure, although its specific role in the clinical benefit of maintenance therapy remains uncertain. Intravesical BCG also induces systemic and local BCG-specific CD4-positive Th1 responses, supporting the contribution of antigen-specific cellular immunity to treatment activity [9].

BCG failure has been associated with an immunosuppressive tumor microenvironment characterized by regulatory T cells, myeloid suppressor populations, macrophage- and fibroblast-rich stromal components, and activation of immune checkpoint pathways [10,11]. These observations provide a rationale for evaluating BCG in combination with agents targeting immune-evasion pathways.

### 3.2. Clinical Categories of BCG Response and Failure

Consistent terminology is required when interpreting studies of treatment response and evaluating therapeutic options after intravesical BCG. BCG-naive disease refers to non-muscle-invasive bladder cancer in patients who have not previously received intravesical BCG. Adequate BCG exposure is generally defined as the administration of at least five of six induction instillations together with at least two of six instillations of a second induction course or at least two of three maintenance instillations [12].

BCG failure is an umbrella term encompassing high-grade disease detected during or after BCG therapy. It includes several clinically distinct patterns with different probabilities of response to further BCG. Low-grade recurrence during or after treatment is not considered BCG failure. BCG-refractory disease refers to persistent high-grade disease despite BCG treatment or the development of a high-grade tumor during maintenance therapy. This category includes T1 high-grade disease detected at the 3-month evaluation, persistent Ta high-grade disease at 3 or 6 months after additional BCG, carcinoma in situ that persists at 6 months after re-induction or the first maintenance course, and high-grade recurrence during BCG maintenance [12,13,14].

BCG-unresponsive disease identifies patients for whom further BCG is unlikely to provide meaningful clinical benefit. It includes BCG-refractory tumors and early high-grade recurrence after adequate BCG exposure. Specifically, this category comprises high-grade Ta or T1 recurrence within 6 months of completing adequate BCG and carcinoma in situ recurrence within 12 months. Radical cystectomy remains the preferred treatment for eligible patients, whereas bladder-preserving approaches may be considered in patients who are unfit for or decline surgery [5,12,15,16].

BCG-exposed disease occupies an intermediate position between BCG-naive and BCG-unresponsive disease. It includes persistent or recurrent high-grade Ta disease or carcinoma in situ at the 3-month evaluation after induction BCG alone, delayed recurrence after an inadequate BCG course, and high-grade recurrence after adequate BCG occurring outside the BCG-unresponsive interval. Patients in this category may still benefit from additional BCG, depending on the timing and pattern of recurrence [14,17,18,19].

The distinction between BCG-refractory and BCG-unresponsive disease is therefore clinically relevant. BCG-refractory disease describes persistent or rapidly recurring high-grade disease during the initial course of BCG treatment, whereas BCG-unresponsive disease is a broader category that includes both refractory disease and early high-grade recurrence after adequate BCG. Consistent application of these definitions is necessary when comparing molecular biomarkers, clinical trial populations, and bladder-preserving treatment strategies [12,14,17,19].

## 4. Molecular Classification of Bladder Cancer: Relevance to BCG Response

### 4.1. Evolution from Single-Gene Markers to Molecular Subtypes

Molecular characterization of bladder tumors has progressed from single-gene analyses to transcriptome-based classification. Early studies focused on assessing the expression of individual genes, such as *S100A8*, *PFKFB4*, *HMOX1*, *MTAP*, *KIF1A*, *COCH*, *CELSR3*, and *MGC1762*, and mutations in the driver genes *FGFR3*, *ERBB2*, *EGFR*, *PIK3CA*, and *TP53*, together with chromosome 9 deletions. In parallel, the identification of the first multigene signatures, in particular *MS1*, *MS2* and *BASE47*, allowed for the prediction of disease stage, presence of regional lymph node metastases and risk of progression [20,21,22,23,24,25,26]. However, the clinical applicability of these early markers remained limited due to low reproducibility of results and insufficient validation in independent patient cohorts.

The introduction of high-throughput sequencing and genome-wide gene expression microarrays has enabled large-scale molecular profiling of tumors. Several molecular classification systems have been proposed for muscle-invasive bladder cancer (classifications of the University of North Carolina, the Broad Institute, MD Anderson, TCGA project, and the Kamoun consensus classification), for non-muscle-invasive bladder cancer (UROMOL2016, UROMOL2021), and for describing both disease variants (Lund, Baylor, T.Z. Tan et al.). The following sections focus on three classification systems with particular relevance to urologic oncology.

### 4.2. UROMOL2021 Classification

In 2021, the results of the European multicenter UROMOL study were published. This study included an integrated multi-omics analysis of 862 non-muscle-invasive bladder tumor samples. The researchers used a combination of whole-transcriptome sequencing, exome analysis, and spatial proteomics to identify four transcriptomic classes, partially overlapping with the three previously described in the UROMOL2016 study [27].

Class 1 is characterized by the expression of early cell cycle genes (*CCND1-3*, *ID1-3*, *RBL2*, *WEE1*) and is often associated with RAS mutations and myeloid dendritic cell infiltration. Tumors in this class generally correspond to the papillary morphology of stage Ta and demonstrate a relatively favorable prognosis. Class 2a is associated with the expression of late cell cycle genes (*CDK1*, *CDK2*, *CDK4*, *CCNE1*, *CDC20*, *CCNB2*, *FOXM1*, and *PLK1*), uroplakins, epithelial differentiation regulators (ESR2, ERBB2, and ERBB3), and genes involved in DNA replication. This class is characterized by frequent TP53 and TSC1 mutations, enrichment in APOBEC mutational signatures, and the highest rate of progression and the worst relapse-free survival. The vast majority of T1HG tumors, as well as cases with concomitant carcinoma in situ, belong to class 2a.

Class 2b is characterized by the expression of cancer stem cell markers and genes involved in epithelial–mesenchymal transition (*AR*, *GATA3*, *ALDH2*, *ALDH1A1*, *CD47*, *FOXF1*, *ZEB1*). Tumors in this class demonstrate the most pronounced immune cell infiltration, and spatial proteomics confirmed an increased density of T lymphocytes in the tumor microenvironment [27].

Class 3 is characterized by co-expression of *FGFR3*-associated genes and *KRT5* and *KRT15*, and a weakened immune response. Tumors in this class typically have activating mutations in *FGFR3* and *PIK3CA*, as well as reduced expression of *AR* and *GATA3*. Prognostically, class 3 occupies an intermediate position between classes 1 and 2.

The UROMOL2021 genomic and transcriptomic subtypes demonstrated independent prognostic value compared with clinical risk factors, including the EORTC score. However, the authors note a significant limitation of the study related to the lack of a standardized treatment protocol. Only 55 patients received BCG therapy, representing less than 7% of the entire sample, and some of these patients only received the induction course. These differences in treatment limited the assessment of molecular signatures specifically associated with BCG response [27].

### 4.3. Broader Molecular Context: TCGA and the Kamoun Consensus

Although The Cancer Genome Atlas (TCGA) and the Kamoun consensus classification were developed primarily for muscle-invasive bladder cancer, they established the main biological axes that are also used to interpret molecular heterogeneity in non-muscle-invasive disease. These systems distinguish luminal, basal-squamous, stromal and neuroendocrine tumor phenotypes and provide a broader context for evaluating transcriptomic classifications associated with BCG response.

The initial TCGA analysis integrated genomic, transcriptomic, epigenetic and proteomic data and demonstrated that urothelial carcinoma is characterized by a high somatic mutation burden, frequent alterations in chromatin-regulating genes and recurrent changes in pathways involving *FGFR3*, *ERBB2*, *TP53*, *RB1* and *PI3K-AKT-mTOR* signaling [2]. The expanded analysis of 412 muscle-invasive tumors subsequently identified five expression subtypes: luminal-papillary, luminal-infiltrated, luminal, basal-squamous and neuronal [28]. Luminal-papillary tumors are associated with *FGFR3* activity, papillary morphology and relatively favorable clinical outcomes. Basal-squamous tumors expressed basal keratins, showed frequent *TP53* and *RB1* alterations and were commonly associated with immune and inflammatory gene expression. The luminal-infiltrated subtype combined luminal differentiation with stromal and immune-cell signatures, whereas the neuronal subtype is characterized by neuroendocrine transcriptional features and an unfavorable prognosis.

Because several independent classification systems used partly overlapping but non-identical terminology, Kamoun et al. integrated transcriptomic data from 1750 muscle-invasive tumors and proposed six consensus molecular classes: luminal papillary, luminal nonspecified, luminal unstable, stroma-rich, basal-squamous and neuroendocrine-like [29]. The luminal papillary class was enriched for *FGFR3* activity and papillary features. Luminal unstable tumors showed high mutation burden and extensive copy-number alterations, including frequent changes involving *TP53*, *ERCC2*, *PPARG* and *E2F3*. The stroma-rich class was defined by fibroblast, smooth-muscle and immune-cell signals, while the basal-squamous class showed basal differentiation, cytotoxic immune infiltration and activation of *STAT3*, *HIF1A* and *EGFR*-associated programs. Neuroendocrine-like tumors lacked prominent immune infiltration and frequently carried concurrent *TP53* and *RB1* alterations.

These classifications cannot be directly transferred to non-muscle-invasive bladder cancer. NMIBC is more frequently associated with papillary morphology, activating *FGFR3* and *PIK3CA* mutations, alterations in chromatin regulators and lower levels of large-scale genomic instability. By contrast, muscle-invasive tumors more commonly show *TP53* and *RB1* pathway disruption, aneuploidy, extensive copy-number alterations and greater intratumoral heterogeneity [30,31,32,33]. Nevertheless, several biological features defined in TCGA and the Kamoun consensus recur in NMIBC classifications. These include *FGFR3*-associated luminal differentiation, basal-squamous transcriptional programs, stromal infiltration, immune-cell enrichment and neuroendocrine features.

The relevance of these broader classifications to BCG therapy is therefore indirect. They help interpret the molecular characteristics of BCG-associated subtypes, including the luminal features of *BRS1* and *BRS2* and the basal-squamous, stromal and immunosuppressive phenotype of *BRS3*. They also provide a common framework for comparing NMIBC-specific systems with datasets originally developed for muscle-invasive disease. However, neither the TCGA nor the Kamoun classification was designed to predict response to intravesical BCG, and neither should be used as an independent basis for treatment selection in NMIBC. Their main value in this setting is biological annotation and cross-cohort comparison rather than direct clinical stratification.

## 5. Candidate Molecular Biomarkers Associated with BCG Response

### 5.1. Transcriptome-Based Classification Systems

#### 5.1.1. BCG Response Subtypes Classification

In 2023, de Jong et al. proposed a transcriptome-based classification of BCG response in Science Translational Medicine. The authors conducted whole-transcriptome sequencing of two independent cohorts of patients with high-risk, non-muscle-invasive bladder cancer. The first cohort included 132 patients who had not previously received BCG therapy and 44 cases of relapse after BCG therapy (34 paired samples before and after treatment), while the validation cohort consisted of 151 patients without prior intravesical treatment. Based on cluster analysis of differential gene expression, three subtypes were identified, designated BCG Response Subtypes (BRS) 1, 2, and 3 [10]. The *BRS1* and *BRS2* subtypes exhibit predominantly luminal properties with expression of the *PPARG* marker and demonstrate a significantly better response to BCG therapy. According to the authors, the treatment response rate was 85% for *BRS1* and 82% for *BRS2* versus 68% for *BRS3* (*p* = 0.017). Two-year progression-free survival was 83% for BRS1, 78% for BRS2, and 61% for BRS3. In multivariable analysis, *BRS3* remained an independent predictor of disease progression.

BRS3 showed the least favorable clinical profile and pronounced basal-squamous features. The molecular profile of these tumors includes increased activity of the epithelial–mesenchymal transition program, enrichment in mutations in extracellular matrix genes, and overexpression of several immunosuppressive molecules. These included immune checkpoint genes (PD-1 and PD-L1), CTLA-4, regulatory T-cell markers (CD4, CD25, and FOXP3), myeloid-derived suppressor cell markers (IL-6, IL-10, CD11b, and CD14), colony-stimulating factors, and chemokines. Spatial proteomics confirmed the depleted immune profile of BRS3 with suppressed cytotoxic T-cell activity in the tumor microenvironment [10].

The study also introduced the BRSpred classifier, based on the nearest shrunken centroid algorithm, and showed that BRSs could be assigned using real-time quantitative PCR. These features may facilitate further clinical evaluation of the classification. Application of the BRS classifier to the UROMOL2021 and TCGA cohorts demonstrated that BRS3 tumors correspond predominantly to UROMOL2021 classes 2a and 2b, as well as the luminal infiltrated and basal-squamous cell subtypes of TCGA. The molecular characteristics of BRS3 provide a rationale for evaluating alternative treatment strategies, including early radical cystectomy and immune checkpoint inhibition, in prospective biomarker-stratified studies.

The association of BRS3 with basal-squamous and immunosuppressive features was observed in both the discovery and validation cohorts reported in the original study [10]. At present, BRS classification should be regarded as a research stratification tool rather than a validated basis for selecting treatment in individual patients.

#### 5.1.2. The MSP888 Signature Predictor

The MSP888 classifier was developed from transcriptomic profiles of 948 patients with non-muscle-invasive bladder cancer. The authors identified three molecular subtypes with different prognoses and responses to BCG therapy [34,35].

The DP.BCG+ subtype is associated with disease progression but demonstrates a positive response to BCG therapy. The REC.BCG+ subtype is associated with poor relapse-free survival but also remains responsive to BCG. The third subtype, designated EP (equivocal prognosis), is characterized by an uncertain prognosis and a less predictable treatment response. Multivariate analysis demonstrated the independent clinical significance of the MSP888 classifier for predicting both disease progression (*p* = 0.001) and relapse (*p* = 0.001). Comparison with the Lund and UROMOL classifications showed that MSP888 more accurately distinguishes patterns of tumor biological activity and BCG response.

Clinical application of MSP888 remains limited by the small number of patients who received both transcriptomic profiling and BCG therapy and by the absence of prospective randomized validation. The classifier therefore remains a candidate for further prospective evaluation and comparison with other molecular platforms.

#### 5.1.3. Transcriptomic Subtypes of Stage T1

Stage T1 tumors carry a substantial risk of progression. A study on the molecular characterization of 73 primary T1 tumors identified five distinct transcriptomic classes: T1-Myc, T1-LumGU, T1-Inflam, T1-TLum, and T1-Early. The T1-Inflam subtype was characterized by the highest immune cell infiltration and a low relapse rate, while T1-Early demonstrated suppression of IFN-α and IFN-γ markers and inflammatory response genes, as well as the lowest immune infiltration [36].

According to the authors, more favorable outcomes after BCG therapy were observed in the T1-LumGU, T1-Inflam, and T1-TLum subtypes, with lower relapse rates in these groups. The T1-Myc and T1-Early subtypes showed the highest median MYC expression and the least favorable recurrence-free survival, indicating that MYC-associated signaling warrants further mechanistic investigation. Limitations of the study include the small sample size, the lack of progression-free survival data, and incomplete information on the details of the administered BCG regimens. The principal transcriptomic classification systems evaluated in non-muscle-invasive bladder cancer and their reported associations with BCG-related outcomes are summarized in Table 1.

### 5.2. Genomic Markers Associated with BCG Response

In addition to comprehensive transcriptomic classifications, individual genomic alterations have demonstrated associations with the efficacy of BCG therapy.

*FGFR3* mutations are frequently found in non-muscle-invasive bladder cancer, particularly in low-grade papillary tumors and stage Ta. However, the independent predictive value of *FGFR3* status for BCG response remains controversial. In current research, *FGFR3* should not be considered an isolated marker of BCG sensitivity or resistance, but rather as a component of a broader molecular context, including stage, grade of malignancy, transcriptomic subtype, immune profile, and associated genomic alterations [27]. Alterations in the p53 pathway are common molecular features of tumor progression in bladder cancer and are associated with a more aggressive biological phenotype. In the integrated multi-omics study UROMOL2021, p53 pathway disruption, high chromosomal instability, and APOBEC-associated mutations were significantly associated with transcriptome class 2a, which is associated with poor clinical prognosis [27]. In the context of predicting response to BCG therapy, NGS panel data indicate the potential value of comprehensive molecular profiling; however, individual mutations, including TP53, cannot be considered yet as independent validated predictors of response [37]. A more consistent predictor of adverse outcome after BCG is the BRS3 integral transcriptome phenotype, characterized by increased activity of the epithelial–mesenchymal transition program, basal and immunosuppressive features, reduced BCG response rate, and worse progression-free survival [10].

Mutations in the telomerase reverse transcriptase (TERT) gene promoter are detected in approximately 56% of tumors treated with the BCG vaccine and are not directly associated with stage or differentiation grade. The rs2853669 polymorphism in TERT is associated with higher-grade tumors, suggesting a potential role for this locus in tumor progression [38]. The value of TERT alterations for predicting BCG response remains uncertain.

APOBEC-mediated mutagenesis is a major source of mutations in urothelial cancer. Tumors with a high APOBEC signature are enriched for mutations in DNA damage response genes (*TP53*, *ATR*, *BRCA2*) and chromatin regulatory genes (*ARID1A*, *MLL*, *MLL3*), while APOBEC-low tumors more often contain *FGFR3* and *KRAS* mutations. A high APOBEC signature is associated with improved overall survival in muscle-invasive disease (38.2 vs. 18.5 months, *p* = 0.005) and, in non-muscle-invasive disease, is enriched in UROMOL2021 class 2a [27,28]. In the context of BCG therapy, data on the prognostic value of the APOBEC signature are contradictory, and its predictive value remains unresolved. Genome-wide association studies (GWASs) identified 12 single-nucleotide polymorphisms (SNPs) that potentially predict intravesical recurrence after BCG therapy. Among them, rs363765 and rs6986852 showed the strongest associations, and a genetic risk model based on these two SNPs discriminated between patients with different risks of intravesical recurrence after BCG therapy, but not after non-BCG intravesical chemotherapy [39]. Polymorphisms in oxidative stress genes, including NEIL2 and UNG, and in inflammation-related genes have also demonstrated associations with recurrence and progression after BCG treatment [40,41].

### 5.3. DNA Methylation Markers

Aberrant DNA methylation is a common epigenetic alteration in bladder carcinogenesis, affecting the promoter regions of tumor suppressor genes in 50–90% of cases. Genes with diagnostically and prognostically relevant aberrant methylation include *TBX2*, *TBX3*, *GATA2*, *ZIC4*, *MGMT*, *RASSF1A*, *TIMP3*, *CDKN2A*, and *CDKN2B* [42].

Genome-wide methylation studies have also compared tumors from BCG responders and nonresponders. Using the Infinium MethylationEPIC BeadChip platform, differential methylation was identified in 40 genomic regions in pretreatment tumors from 26 BCG responders and 27 patients with BCG failure. Six regions located in the promoters of *GPR158*, *KLF8*, *C12orf42*, *WDR44*, *FLT1*, and *CHST11* were subsequently validated by bisulfite sequencing. *GPR158* promoter hypermethylation showed the strongest discrimination for BCG failure in this cohort, with an area under the receiver operating characteristic curve of 0.809 (*p* < 0.001) [43]. Because the study lacked external validation, *GPR158* methylation should be regarded as a candidate biomarker rather than an established predictor of BCG response.

In a retrospective study of 91 patients with primary T1G3 non-muscle-invasive bladder cancer treated with induction BCG without maintenance, methylation of several tumor suppressor genes was associated with clinical outcome. A two-gene model based on *MSH6* and *THBS1* methylation provided the strongest independent assessment of progression risk, whereas *GATA5* methylation was associated with both progression and disease-specific survival [44]. These findings have not been prospectively validated and do not establish the ability of these markers to select patients for BCG or alternative treatment.

In a prospective multicenter cohort of 1239 patients with non-muscle-invasive bladder cancer, *FGFR3* mutation was associated with a lower risk of progression, whereas *GATA2* and *TBX3* methylation were associated with a higher risk. Combining *FGFR3* mutation status with *GATA2* methylation improved progression-risk stratification within the EAU high-risk group and separated patients into groups with lower, intermediate, and higher progression incidence rates [45]. This model was developed for prognostic refinement and was not designed to predict response to BCG specifically.

Methylation-based analyses have also identified bladder cancer subgroups differing in HLA and MHC class I expression, antigen-presenting-cell co-stimulatory and co-inhibitory programs, immune checkpoint activity, and type I and type II interferon-response signatures [46]. These associations suggest that the epigenetic state of the tumor may contribute to variation in the immune microenvironment, but a direct relationship between these methylation patterns and the efficacy of intravesical BCG has not been established.

### 5.4. Tumor Microenvironment and Immune Profile

The tumor microenvironment contributes to variation in BCG response because the treatment depends on activation of cellular immunity within the bladder mucosa. Tumor immunophenotyping and cytokine profiling have therefore been evaluated as potential stratification methods.

In a study of 40 BCG-treated patients, low densities of CD4-positive and GATA3-positive T cells and increased densities of FOXP3-positive and CD25-positive regulatory T cells and CD68-positive and CD163-positive macrophages were associated with BCG failure [47]. Experimental studies support a central role for Th1-associated cytokine signaling in BCG-induced immunity, but the clinical predictive value of individual urinary or tissue cytokines remains uncertain [48]. In a small clinical study, urinary Th1 cytokine profiles, including IFN-γ and IL-2, were associated with response to BCG, but the limited sample size precludes their use as validated clinical biomarkers [49].

Exhausted CD8-positive T-cell populations may also be associated with BCG response. In a prospective analysis of 27 patients with non-muscle-invasive bladder cancer, a subpopulation of intratumoral CD8-positive T cells co-expressing PD-1, CD38, and Tim-3 was associated with an unfavorable response to BCG therapy. These cells exhibit a reduced ability to produce IFN-γ and granzyme B, reflecting their functional exhaustion [50]. These populations warrant further evaluation as biomarkers in trials of BCG combined with immune checkpoint inhibitors.

Baseline PD-L1 expression has been evaluated as a candidate biomarker of BCG failure. In one study, PD-L1 positivity was observed in 25–28% of BCG nonresponders and 0–4% of responders, with PD-L1-positive cells colocalizing with CD8-positive T cells [51]. However, a larger subsequent study found no association between pretreatment PD-L1 expression and BCG failure or progression-free survival [52]. Differences in antibody clones, scoring methods, cutoffs, timing of tissue collection, and cohort composition limit cross-study comparison.

### 5.5. Liquid-Biopsy Biomarkers

Liquid-biopsy approaches in non-muscle-invasive bladder cancer primarily involve the analysis of urine and, less frequently, peripheral blood. Candidate urinary biomarkers include exfoliated tumor cells, tumor-derived DNA and RNA, proteins, and soluble immune mediators. Blood-based analyses have examined circulating tumor DNA, cytokines, and circulating immune-cell populations. Serial sampling allows molecular and immunological changes to be assessed before, during, and after intravesical treatment.

The intended clinical use of a biomarker should be defined before its evaluation. Diagnostic biomarkers detect the presence of disease, whereas prognostic biomarkers identify differences in the risk of recurrence, progression, or death irrespective of treatment. Monitoring biomarkers reflect changes in tumor burden or treatment-induced biological responses over time. Treatment-response predictive biomarkers identify differences in the benefit of a specific therapy according to biomarker status and therefore require comparison between treatment groups.

Post-BCG T-cell exhaustion and urinary tumor DNA levels have been associated with high-grade recurrence and BCG failure [53]. Along with the urinary cytokine profiles described above, these findings support potential monitoring and prognostic applications. They do not establish treatment-response predictive value because differential benefit from BCG and alternative treatments was not assessed according to biomarker status.

Collectively, available studies indicate that BCG response is associated with a composite molecular and immune phenotype rather than with a single marker. Luminal differentiation, immune-cell composition, chromosomal stability, and the expression of PD-L1, MYC, and immunosuppressive genes have each been associated with outcomes, although findings vary across cohorts. These features require validation in standardized prospective studies before they can guide treatment selection. Transcriptome profiling may also identify therapeutic targets in BCG-naive and BCG-refractory non-muscle-invasive bladder cancer.

## 6. Current and Emerging Therapeutic Strategies in the BCG Setting

Treatment selection in high-risk non-muscle-invasive bladder cancer is determined primarily by previous BCG exposure, the timing and pattern of recurrence, the presence of carcinoma in situ, and patient eligibility for radical cystectomy. BCG-naive and BCG-unresponsive disease represent distinct clinical settings and should be considered separately. Molecular alterations may identify patients for biomarker-stratified trials, but they do not currently provide a validated basis for routine treatment allocation.

### 6.1. BCG-Naive High-Risk Disease: Combination Strategies

BCG induction followed by maintenance remains the treatment backbone for BCG-naive high-risk non-muscle-invasive bladder cancer. The activation of the PD-1/PD-L1 axis during or after BCG treatment has prompted evaluation of systemic immune checkpoint inhibitors in combination with intravesical BCG. However, PD-L1 expression and other immune biomarkers have not been validated as criteria for selecting patients for these combinations.

The phase III CREST study evaluated subcutaneous sasanlimab, a monoclonal antibody against PD-1, in combination with BCG in 1055 patients with BCG-naive high-risk non-muscle-invasive bladder cancer. Patients were randomized to receive sasanlimab with BCG induction and maintenance, sasanlimab with BCG induction alone, or BCG induction and maintenance. The primary comparison between sasanlimab with BCG induction and maintenance and BCG alone demonstrated an improvement in event-free survival, with a hazard ratio of 0.68 (95% CI 0.49–0.94; one-sided *p* = 0.0095). The estimated 36-month event-free survival rates were 82.1% and 74.8%, respectively [54].

The phase III POTOMAC study evaluated durvalumab combined with BCG in 1018 patients with BCG-naive high-risk disease. Durvalumab was administered every four weeks for 13 cycles together with BCG induction and maintenance. The combination reduced the risk of high-risk disease recurrence or death compared with BCG induction and maintenance alone (HR 0.68, 95% CI 0.50–0.93; *p* = 0.0154). Grade 3 or 4 treatment-related adverse events occurred in 21% of patients receiving durvalumab with BCG induction and maintenance and in 4% of those receiving BCG alone [55]. On 28 May 2026, the FDA approved durvalumab in combination with BCG for adults with BCG-naive high-risk non-muscle-invasive bladder cancer [56].

In contrast, the phase III ALBAN study did not demonstrate a clinical benefit from adding atezolizumab to BCG. The study randomized 517 BCG-naive patients to BCG induction and maintenance with or without intravenous atezolizumab. Event-free survival did not differ between the groups (HR 0.98, 95% CI 0.71–1.36; *p* = 0.9106), whereas treatment-related adverse events, including events of grade 3 or higher, were more frequent with combination therapy [57].

The phase III KEYNOTE-676 study is evaluating pembrolizumab combined with BCG in patients with persistent or recurrent high-risk disease after BCG induction and in a separate cohort of BCG-naive patients. Participants receive pembrolizumab with BCG or BCG alone. At the time of the literature search, the study was active but no longer recruiting, and efficacy results had not been posted. The clinical role of pembrolizumab combined with BCG in these populations therefore remains unresolved [58].

The contrasting results of CREST, POTOMAC, and ALBAN indicate that the effect of adding immune checkpoint inhibition to BCG cannot be considered uniform across the drug class. Differences in the checkpoint inhibitor, route and duration of treatment, BCG maintenance schedule, patient population, and study design may contribute to variation in efficacy. Biomarker analyses are required to determine whether immune or molecular characteristics identify patients who benefit from a specific combination. The principal randomized phase III studies of immune checkpoint inhibitors combined with BCG are summarized in Table 2.

### 6.2. FDA-Approved Bladder-Preserving Options for BCG-Unresponsive Disease

Radical cystectomy remains the preferred oncological treatment for eligible patients with BCG-unresponsive high-risk non-muscle-invasive bladder cancer. Radical cystectomy remains the preferred oncological treatment for eligible patients with BCG-unresponsive high-risk non-muscle-invasive bladder cancer [5,16]. Bladder-preserving treatment may be considered when radical cystectomy is contraindicated or declined. Several systemic and intravesical therapies have received FDA approval for selected patients, primarily those with carcinoma in situ with or without papillary tumors.

Pembrolizumab is a systemic monoclonal antibody against PD-1. In cohort A of the phase II KEYNOTE-057 study, 101 patients with BCG-unresponsive carcinoma in situ, with or without papillary disease, received pembrolizumab after declining or being considered ineligible for radical cystectomy. The complete response rate at approximately 3 months was 41%, and the median duration of response was 16.2 months. These results formed the basis for FDA approval in January 2020 for patients with BCG-unresponsive high-risk non-muscle-invasive bladder cancer with carcinoma in situ, with or without papillary tumors, who are ineligible for or have elected not to undergo cystectomy [59,60].

Nadofaragene firadenovec-vncg (Adstiladrin) is a non-replicating adenoviral vector-based gene therapy encoding interferon alpha-2b. In the phase III CS-003 study, 53.4% of patients with carcinoma in situ, with or without a concomitant high-grade papillary tumor, achieved a complete response within 3 months. Among responders, 45.5% maintained the response for 12 months [61]. The FDA approved nadofaragene firadenovec in December 2022 for adults with high-risk BCG-unresponsive non-muscle-invasive bladder cancer with carcinoma in situ, with or without papillary tumors [62]. Five-year follow-up confirmed prolonged bladder preservation in a proportion of treated patients, although durable high-grade recurrence-free survival declined over time [63].

Nogapendekin alfa inbakicept-pmln (Anktiva) is an IL-15 superagonist protein complex administered intravesically in combination with BCG. In QUILT-3.032, the FDA-evaluable population included 77 patients with BCG-unresponsive carcinoma in situ, with or without high-grade Ta or T1 papillary disease. The complete response rate was 62%; 58% of responders maintained a response for at least 12 months, and 40% maintained a response for at least 24 months. The combination received FDA approval in April 2024 for adults with BCG-unresponsive non-muscle-invasive bladder cancer with carcinoma in situ, with or without papillary tumors [64,65].

The gemcitabine intravesical system, initially developed as TAR-200 and marketed in the United States as Inlexzo, provides sustained local delivery of gemcitabine through a device placed within the bladder. Cohort 2 of the phase IIb SunRISe-1 study included 83 patients with BCG-unresponsive carcinoma in situ, with or without papillary tumors. The complete response rate was 82%, and 51% of responders maintained a response for at least 12 months [66]. The FDA approved the gemcitabine intravesical system in September 2025 for this population [67].

These treatments differ in route of administration, treatment schedule, mechanism of action, toxicity profile, and duration of follow-up. Their efficacy estimates were derived mainly from separate single-arm studies with different assessment schedules and definitions. Complete response rates should therefore not be used for direct comparative ranking in the absence of randomized head-to-head trials.

### 6.3. Investigational Therapies for BCG-Unresponsive Disease

#### 6.3.1. Investigational Immune Checkpoint Inhibitors

Atezolizumab was evaluated as systemic monotherapy in the phase II SWOG S1605 study in patients with BCG-unresponsive carcinoma in situ, with or without papillary tumors, who were ineligible for or declined radical cystectomy. The complete response rate at 6 months was 27%, and 56% of patients who achieved a complete response maintained it for at least 12 months. The study did not meet its prespecified efficacy threshold and did not support regulatory approval of atezolizumab for this indication [68].

Cetrelimab, a monoclonal antibody against PD-1, was evaluated as monotherapy in cohort 3 of the phase IIb SunRISe-1 study. Cetrelimab produced a confirmed complete response rate of 46.4%, with an estimated complete response rate of 22.8% at 12 months. The cetrelimab and gemcitabine intravesical system cohorts were parallel study cohorts rather than a randomized head-to-head comparison. Differences in response rates between them should therefore be interpreted cautiously [69].

#### 6.3.2. Investigational Intravesical and Antibody-Directed Therapies

Cretostimogene grenadenorepvec, formerly designated CG0070, is an oncolytic adenovirus encoding granulocyte-macrophage colony-stimulating factor. It preferentially replicates in tumor cells with defective retinoblastoma pathway activity. The phase III BOND-003 study is evaluating intravesical cretostimogene in patients with BCG-unresponsive carcinoma in situ, with or without papillary tumors, and in a separate cohort with papillary high-grade Ta or T1 disease without carcinoma in situ. Interim analyses have reported a complete response rate of approximately 75% in the carcinoma in situ cohort, with 46% of responders maintaining a complete response for at least 12 months. These findings remain preliminary, and the treatment has not received FDA approval for this indication [70,71].

Detalimogene voraplasmid, previously designated EG-70, is a non-viral intravesical gene therapy designed to induce local expression of immune-stimulatory proteins within the bladder urothelium. The phase I/II LEGEND study includes patients with BCG-unresponsive carcinoma in situ, BCG-exposed or BCG-naive high-risk disease, and BCG-unresponsive papillary tumors without carcinoma in situ. Preliminary analyses in BCG-unresponsive carcinoma in situ have reported complete response rates of 68% at 3 months and 45% at 6 months. The study remains ongoing, and longer follow-up is required to assess response durability and clinical safety [70,72,73].

TARA-002 is an intravesical immunotherapy derived from a modified Streptococcus pyogenes preparation. The phase II ADVANCED-2 study includes separate cohorts of patients with BCG-naive, BCG-exposed, and BCG-unresponsive carcinoma in situ, with or without papillary disease. Interim findings indicate clinical activity, but the number of evaluable patients remains limited and long-term response data are not yet available [74,75].

Oportuzumab monatox, also known as Vicinium, is an EpCAM-directed recombinant fusion protein containing a truncated fragment of Pseudomonas aeruginosa exotoxin A. In a phase II study of 46 patients with BCG-refractory carcinoma in situ, complete response rates at 3 months were 41% and 39% in the two treatment cohorts [76]. Oportuzumab monatox was subsequently evaluated in the single-arm phase III VISTA study [77], but did not receive FDA approval and should not be presented as an available standard treatment.

Disitamab vedotin (RC48) is a HER2-directed antibody–drug conjugate comprising an anti-HER2 monoclonal antibody linked to monomethyl auristatin E. A phase I study evaluated intravesical disitamab vedotin in patients with HER2-expressing high-risk non-muscle-invasive bladder cancer and reported preliminary evidence of feasibility and antitumor activity. The small dose-escalation cohort does not allow definitive assessment of efficacy [78]. Further studies are evaluating intravesical disitamab vedotin and systemic disitamab vedotin combined with BCG in patients with HER2-expressing high-risk disease [79,80].

### 6.4. Molecularly Selected Targeted Therapy

FGFR3 alterations are frequent in papillary non-muscle-invasive bladder cancer and represent one of the most clearly defined molecular targets in this disease. Cohort 1 of the phase II THOR-2 study evaluated oral erdafitinib in patients with recurrent, papillary-only high-risk non-muscle-invasive bladder cancer harboring selected FGFR2 or FGFR3 alterations after previous BCG treatment. Patients were ineligible for or declined radical cystectomy and were randomized to erdafitinib or investigator-selected intravesical chemotherapy.

The study randomized 73 patients before enrollment was discontinued because of slow accrual. Erdafitinib prolonged recurrence-free survival compared with intravesical mitomycin C or gemcitabine. Median recurrence-free survival was not reached in the erdafitinib group and was 11.6 months in the intravesical chemotherapy group, with an estimated hazard ratio of 0.28 (95% CI 0.10–0.60; nominal *p* = 0.0008) [81].

Despite these findings, erdafitinib is not approved for non-muscle-invasive bladder cancer. Its approved urothelial carcinoma indication applies to selected patients with locally advanced or metastatic FGFR3-altered disease. In NMIBC, FGFR testing may identify eligibility for clinical trials but does not currently provide a routine indication for FGFR inhibitor treatment.

Other genomic and molecular features, including APOBEC mutational signatures, transcriptomic BRS status, GPR158 methylation, germline polymorphisms, and PD-L1 expression, have not been prospectively validated as treatment-selection biomarkers. Their potential value lies in prospective biomarker-stratified studies rather than in routine assignment of patients to BCG, immune checkpoint inhibition, gene therapy, intravesical chemotherapy, or radical cystectomy.

## 7. Clinical Integration of Molecular Data: Current Limits and Prospective Framework

Current treatment selection in non-muscle-invasive bladder cancer remains based primarily on clinical and pathological assessment. Tumor stage and grade, the presence of concomitant carcinoma in situ, tumor size and multifocality, recurrence history, previous intravesical treatment, adequacy of BCG exposure, the category of BCG failure, eligibility for radical cystectomy, and patient preferences determine the management strategy. The EAU risk classification incorporates several of these variables and assigns patients to low-, intermediate-, high-, or very-high-risk groups [5,82].

The 2026 EAU Guidelines introduced the option of adding sasanlimab or durvalumab to BCG induction and maintenance in selected, well-informed patients with BCG-naive high- or very-high-risk disease. This recommendation was based on the positive results of the phase III CREST and POTOMAC trials [54,55]. In May 2026, the FDA approved durvalumab in combination with BCG for adults with BCG-naive high-risk non-muscle-invasive bladder cancer [56]. These developments expand the available treatment options but do not establish a molecularly selected approach, because neither combination is currently assigned on the basis of a validated tumor biomarker.

Transcriptomic classifications may add biological and prognostic information to conventional risk assessment. UROMOL2021 identifies molecular classes associated with different risks of recurrence and progression, whereas BRS, MSP888, and the stage T1 transcriptomic classification have shown associations with outcomes after BCG treatment [10,27,34,35,36]. However, none of these systems has been prospectively shown to improve outcomes when used to assign individual patients to BCG, immune checkpoint inhibition, radical cystectomy, or another treatment. Their analytical reproducibility across platforms, clinically relevant cutoffs, and added value over established clinicopathological models also remain insufficiently defined.

Individual genomic, epigenetic, and immune markers are subject to similar limitations. *FGFR2* or *FGFR3* alterations may identify patients for trials of *FGFR*-directed therapy, particularly in recurrent papillary disease, but erdafitinib is not approved for non-muscle-invasive bladder cancer [81]. *APOBEC* mutational signatures, *TP53* alterations, *PD-L1* expression, *GPR158* methylation, germline polymorphisms, and immune-cell composition have been associated with clinical outcomes in individual cohorts, but their predictive value has not been confirmed in prospective treatment-selection studies [28,37,39,43,51].

**Figure 1:**
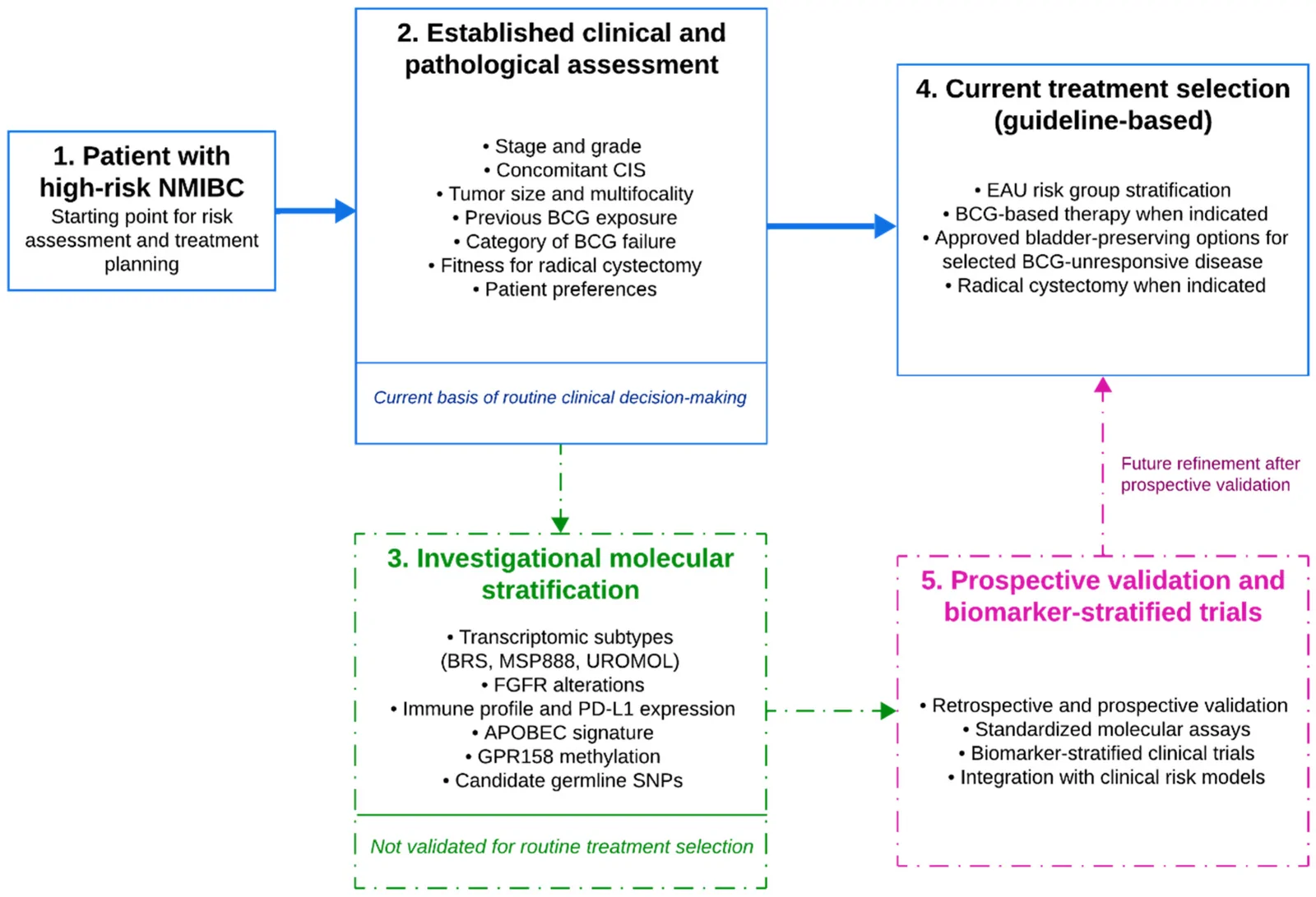
Conceptual framework for the prospective integration of molecular data into treatment stratification for high-risk non-muscle-invasive bladder cancer. Established clinical and pathological factors remain the basis of treatment selection. Transcriptomic classifications, genomic alterations, immune profiles, and epigenetic markers are presented as investigational layers that may refine risk stratification after prospective validation. Solid arrows indicate the current guideline-based clinical pathway, whereas dashed arrows indicate potential future integration of molecular data.

Molecular profiling should therefore be regarded as an investigational layer that may complement, but not replace, established clinical assessment. Its current roles include biological characterization, retrospective risk analysis, identification of eligibility for biomarker-specific trials, and stratification within prospective studies. The distinction between the established clinical pathway and the potential future contribution of molecular data is shown in Figure 1.

Figure 1 should not be interpreted as a validated treatment algorithm. In particular, *BRS3* status alone should not be used to recommend early radical cystectomy or immune checkpoint inhibition. Likewise, the detection of an *FGFR* alteration does not constitute a routine indication for *FGFR* inhibitor treatment in non-muscle-invasive disease, and *PD-L1* expression, *APOBEC* activity, *GPR158* methylation, or candidate germline variants should not independently determine treatment selection.

Prospective evidence must demonstrate that adding molecular information to clinicopathological assessment changes treatment decisions and improves clinically relevant outcomes. Until such evidence is available, molecular testing should be used mainly within clinical trials or structured translational studies. Decisions concerning repeat BCG, bladder-preserving therapy, or radical cystectomy should continue to follow current clinical definitions and guideline-based risk assessment.

## 8. Future Directions and Requirements for Clinical Validation

### 8.1. Study Design and Predictive Biomarker Validation

The transition from molecular association to clinically useful prediction requires prospective validation in clearly defined patient populations. Most molecular studies of BCG response have been retrospective and have included heterogeneous treatment regimens, variable definitions of adequate BCG exposure, and different clinical endpoints.

Prognostic and predictive biomarkers should be distinguished explicitly. A prognostic biomarker is associated with recurrence, progression, or survival irrespective of the treatment received. A predictive biomarker identifies differences in the effect of a specific treatment according to biomarker status. An association between a molecular feature and recurrence within a cohort treated with BCG does not establish predictive utility, because the same feature may be associated with an unfavorable outcome regardless of treatment.

Studies intended to establish predictive value should prespecify the biomarker hypothesis, assay platform, analytical cutoff, treatment groups, and statistical analysis of the biomarker–treatment interaction. Randomized comparative trials provide the most direct assessment of whether treatment effects differ between biomarker-defined groups. When randomization is not feasible, prospective comparative designs should include an appropriate treatment comparator and adjustment for established clinical and pathological prognostic factors. A biomarker-guided strategy should ultimately be compared with treatment selection based on standard clinicopathological assessment.

Biomarker-stratified trials could evaluate BRS-based selection in studies of BCG combinations, *FGFR2* or *FGFR3* alterations in trials of FGFR-directed therapy, HER2 expression in studies of antibody–drug conjugates, and immune profiles in trials of checkpoint inhibitors [10,78,79,80,81]. These designs must determine whether a marker predicts benefit from a particular treatment rather than merely identifies tumors with a different baseline prognosis.

### 8.2. Clinical Information, Biospecimen, and Assay Standardization

Clinical information and treatment exposure should be recorded using uniform criteria. Relevant variables include tumor stage and grade, concomitant carcinoma in situ, tumor size and multifocality, recurrence history, previous intravesical treatment, the number and timing of BCG instillations, completion of induction and maintenance courses, and the interval between treatment and recurrence. Definitions of adequate BCG exposure, BCG-exposed disease, and BCG-unresponsive disease should be specified before enrollment and applied consistently throughout the study.

Biospecimen collection and analytical procedures also require standardization. Relevant preanalytical variables include the timing of sampling in relation to transurethral resection and BCG treatment, the use of primary or recurrent tumors, fixation and storage conditions, tumor cellularity, and RNA and DNA quality. Analytical variables include sequencing depth, normalization procedures, correction for batch effects, assay reproducibility, and the definition of clinically relevant thresholds. Classifiers developed from fresh-frozen tissue or whole-transcriptome sequencing should be converted into reproducible assays suitable for formalin-fixed, paraffin-embedded specimens before routine implementation can be considered.

The temporal and spatial heterogeneity of the tumor microenvironment presents an additional challenge. A single pretreatment specimen may not represent immune and molecular changes that develop during BCG therapy. Longitudinal analysis of tumor tissue, urine, blood, and exfoliated urothelial cells may help distinguish stable tumor characteristics from treatment-induced changes. Spatial proteomics, multiplex immunofluorescence, single-cell sequencing, and spatial transcriptomics may further define the cellular organization of BCG-responsive and BCG-resistant tumors. Spatial immune profiling and an imaging mass cytometry-based classifier have identified immune and stromal features associated with BCG failure, although these findings remain exploratory and require validation in larger independent cohorts [11].

### 8.3. Endpoint Selection and Clinical Implementation

Clinical endpoints should be defined prospectively and harmonized across studies. Complete response at 3 or 6 months is relevant in carcinoma in situ but does not fully characterize long-term clinical benefit. Studies should include duration of response, high-grade recurrence-free survival, progression to muscle-invasive disease, cystectomy-free survival, metastasis-free survival, overall survival, treatment-related toxicity, and patient-reported outcomes. The timing of assessments and the criteria used to define recurrence, progression, and treatment failure should be specified in the study protocol.

Machine learning methods may be applied to clinical, pathological, imaging, and molecular data. As a clinicopathological example, the PROGRxN-BCa model was developed using 14 clinical and pathological features and externally evaluated in 9335 patients. It achieved a concordance index of 0.79 and outperformed the EAU risk calculator for predicting progression [83]. Despite this external evaluation, prospective impact studies are required to determine whether implementation of the model improves treatment decisions and patient outcomes.

Clinical implementation also depends on feasibility. Turnaround time, tissue requirements, cost, access to sequencing, interpretability of results, and the availability of biomarker-specific treatment options influence routine use. Ongoing limitations in BCG availability increase the potential value of identifying patients most likely to benefit, but molecularly guided allocation of BCG requires prospective clinical and economic evaluation. A classifier should not be introduced into routine care unless its use improves treatment selection or patient outcomes beyond established clinicopathological assessment.

## 9. Conclusions

Bladder cancer is characterized by a high somatic mutation burden and frequent alterations in chromatin-modifying genes. Although BCG remains a standard adjuvant treatment for intermediate- and high-risk non-muscle-invasive bladder cancer, a substantial proportion of patients experience treatment failure. Current clinical and pathological risk models have limited ability to predict individual response.

Transcriptomic classifications, including UROMOL2021, BRS, MSP888, and the molecular subtypes described for stage T1 disease, have identified tumor phenotypes associated with recurrence, progression, and BCG response. Genomic alterations, DNA methylation patterns, and features of the tumor immune microenvironment have shown associations with clinical outcomes and may provide additional prognostic information. Their value as treatment-specific predictive biomarkers remains unproven.

FDA-approved bladder-preserving options for selected patients with BCG-unresponsive non-muscle-invasive bladder cancer include pembrolizumab, nadofaragene firadenovec, nogapendekin alfa inbakicept-pmln combined with BCG, and the gemcitabine intravesical system. Cretostimogene grenadenorepvec, detalimogene voraplasmid, TARA-002, oportuzumab monatox, and disitamab vedotin remain investigational or non-approved in this setting. Their integration with molecular profiling requires prospective studies using standardized definitions, treatment regimens, molecular assays, and clinical endpoints. Until such evidence becomes available, molecular classifications should be regarded primarily as research and trial-stratification tools rather than replacements for established clinical risk assessment.

## Figures and Tables

**Table 1:** Major transcriptomic classification systems evaluated in non-muscle-invasive bladder cancer and their relevance to BCG response.

Classification	Study Population	Principal Subtypes	Association with BCG Outcome	Main Limitation	Current Clinical Status
UROMOL2021	862 NMIBC tumors included in a multicenter multi-omics study. Only 55 patients received BCG, and treatment regimens were not standardized.	Classes 1, 2a, 2b, and 3	Class 2a was associated with the highest progression risk and the shortest recurrence-free survival. Class 2b showed marked immune infiltration. A BCG-specific predictive association was not established.	Small and clinically heterogeneous BCG-treated subgroup; variable induction and maintenance exposure; the study was not designed specifically to predict BCG response.	Prognostic and biological classification. Not validated for BCG treatment selection.
BCG Response Subtype (BRS)	High-risk NMIBC analyzed in discovery and validation cohorts, including pretreatment tumors and paired samples obtained before and after BCG failure.	BRS1, BRS2, and BRS3	BCG response rates were 85% for BRS1, 82% for BRS2, and 68% for BRS3. Two-year progression-free survival was 83%, 78%, and 61%, respectively. BRS3 remained an independent predictor of progression.	Retrospective classification; treatment allocation was not based on subtype; no prospective biomarker-guided trial has demonstrated clinical benefit.	Research stratification tool. BRSpred and quantitative PCR provide potential platforms for prospective validation but are not approved for routine treatment assignment.
MSP888	Transcriptomic profiles from 948 patients with NMIBC, with outcome analyses performed in a smaller subset of BCG-treated patients.	DP.BCG+, REC.BCG+, and EP	DP.BCG+ was associated with progression but retained apparent BCG responsiveness. REC.BCG+ was associated with poor recurrence-free survival but also showed BCG response. EP had an equivocal prognosis and less predictable treatment response.	Limited number of patients with both transcriptomic profiling and documented BCG treatment; absence of prospective randomized validation.	Investigational classifier requiring prospective validation and comparison with other molecular platforms.

Abbreviations: BCG, Bacillus Calmette–Guérin; NMIBC, non-muscle-invasive bladder cancer; BRS, BCG Response Subtype; DP, disease progression; REC, recurrence; EP, equivocal prognosis.

**Table 2:** Randomized phase III trials of systemic immune checkpoint inhibitors combined with BCG in high-risk non-muscle-invasive bladder cancer.

Clinical Trial	Agent and Treatment Regimen	Study Population	Main Efficacy Outcome	Safety Findings	Trial Interpretation and Current Status
CREST (NCT04165317) [54]	Subcutaneous sasanlimab plus BCG induction and maintenance; subcutaneous sasanlimab plus BCG induction alone; or BCG induction and maintenance	BCG-naive high-risk NMIBC; 1055 patients in the randomized BCG-naive cohort	Sasanlimab plus BCG induction and maintenance improved EFS compared with BCG induction and maintenance alone: HR 0.68 (95% CI 0.49–0.94; one-sided *p* = 0.0095). Estimated 36-month EFS was 82.1% versus 74.8%.	The safety profile was consistent with the known toxicities of sasanlimab and BCG. Immune-mediated adverse events were more frequent with combination treatment than with BCG alone.	Positive. Sasanlimab plus BCG induction and maintenance is included as an option for selected BCG-naive patients in the 2026 EAU Guidelines. Sasanlimab has not received FDA approval for this indication.
POTOMAC (NCT03528694) [55]	Intravenous durvalumab every 4 weeks for 13 cycles plus BCG induction and maintenance; durvalumab plus BCG induction alone; or BCG induction and maintenance	BCG-naive high-risk NMIBC; 1018 patients	Durvalumab plus BCG induction and maintenance improved DFS compared with BCG induction and maintenance alone: HR 0.68 (95% CI 0.50–0.93; *p* = 0.0154). Median DFS was not reached in either group.	Grade 3 or 4 treatment-related adverse events occurred in 21% of patients receiving durvalumab plus BCG induction and maintenance and 4% receiving BCG alone.	Positive. Durvalumab plus BCG is included in the 2026 EAU Guidelines and was approved by the FDA on 28 May 2026 for adults with BCG-naive high-risk NMIBC.
ALBAN/GETUG-AFU 37 (NCT03799835) [57]	Intravenous atezolizumab 1200 mg every 3 weeks for up to 1 year plus BCG induction and maintenance, or BCG induction and maintenance alone	BCG-naive high-risk NMIBC; 517 patients	No improvement in EFS: HR 0.98 (95% CI 0.71–1.36; *p* = 0.9106). Results were consistent across prespecified subgroups.	Treatment-related adverse events and grade 3 or higher treatment-related adverse events were more frequent with atezolizumab plus BCG.	Negative. The trial did not support the addition of atezolizumab to BCG in this setting.

Abbreviations: BCG, Bacillus Calmette–Guérin; DFS, disease-free survival; EAU, European Association of Urology; EFS, event-free survival; FDA, US Food and Drug Administration; HR, hazard ratio; NMIBC, non-muscle-invasive bladder cancer.

## Data Availability

No new data were created or analyzed in this study. Data sharing is not applicable to this article.
